# Treatment with omega-3 PUFAs does not increase the risk of CYP2E1-dependent oxidative stress and diabetic liver pathology

**DOI:** 10.3389/fendo.2022.1004564

**Published:** 2022-09-26

**Authors:** Oksana Maksymchuk, Angela Shysh, Dmytro Stroy

**Affiliations:** ^1^ Department of Molecular Oncogenetics, Institute of Molecular Biology and Genetics, National Academy of Sciences of Ukraine, Kyiv, Ukraine; ^2^ Department of General and Molecular Pathophysiology, Bogomoletz Institute of Physiology, National Academy of Sciences of Ukraine, Kyiv, Ukraine

**Keywords:** omega-3 PUFAs, CYP2E1, oxidative stress, diabetes, liver

## Abstract

An increase in CYP2E1 expression is a key factor in the development of diabetic oxidative liver damage. Long-term treatment with omega-3 PUFAs, which are CYP2E1 substrates, may affect CYP2E1 expression in the liver. In this work, we performed Western blot analysis, biochemical methods, and microscopic ultrastructural studies of the liver in a streptozotocin-induced rat model of type 1 diabetes to investigate whether long-term treatment with omega-3 PUFAs could induce CYP2E1-dependent oxidative stress and diabetic liver pathology. Significant hyperglycemia and lack of natural weight gain were observed in the diabetic rats compared to non-diabetic controls. A 2.5-fold increase in CYP2E1 expression (protein content and activity) was also observed in the diabetic rats. In addition, signs of oxidative stress were found in the liver of the diabetic rats. A significant increase in transaminases and GGT level in blood serum was also observed, which could indicate marked destruction of liver tissue. Diabetic dyslipidemia (increased triacylglycerol levels and decreased HDL-C levels) was found. Treatment of the diabetic animals with an omega-3-enriched pharmaceutical composition of PUFAs had no effect on CYP2E1 levels but contributed to a two-fold decrease in enzyme activity. The intensity of lipid peroxidation also remained close to the diabetic group. However, at the same time, antioxidant protection was provided by induction of antioxidant enzyme activity. Examination of the liver ultrastructure revealed no characteristic signs of diabetic pathology. However, omega-3 PUFAs did not normalize blood glucose levels and serum lipid profile. Thus, long-term treatment of diabetic rats with omega-3 PUFAs does not increase the risk of CYP2E1-dependent oxidative stress and development of liver pathology but prevents some diabetic ultrastructural damage to hepatocytes.

## Introduction

Diabetes mellitus is one of the most common endocrine diseases in people of all ages around the world. Today, the problem of widespread prevalence of this disease among young people is particularly relevant. Diabetes mellitus is the cause of development and progression of pathology of systems and organs, including liver. In patients with type 2 diabetes mellitus, severe liver disease is often the cause of death (1). Unlike type 2 diabetes, type 1 diabetic liver disease develops slowly and is often asymptomatic. At the same time, an increase in liver-specific enzymes (gamma-glutamyl transferase (GGT), alanine aminotransferase (ALT), aspartate aminotransferase (AST)) is detected in the blood serum of patients, which may indicate the onset of liver pathology (2). The development of liver pathology in type 1 diabetics has been demonstrated in animal models, particularly in our recent work, which showed a sharp increase in liver-specific enzymes in blood serum, significant ultrastructural disturbances, and evidence of oxidative stress in the liver (3).

It is well known that oxidative stress is one of the main mechanisms for the development of diabetes mellitus. It has been shown that cytochrome P450 2E1 (CYP2E1) is the main source of reactive oxygen species (ROS) formation in the liver and that an increase in the expression of this enzyme is one of the key factors for the development of oxidative stress in the liver (4). It was found that oxidative stress mediated by the ROS generated by CYP2E1 damages hepatocytes by peroxidation of cellular macromolecules, namely, lipid peroxidation, protein carbonylation, and oxidative damage to DNA. This leads to an increase in apoptotic processes in hepatocytes and liver fibrosis. CYP2E1-dependent oxidative processes can lead to inhibition of lipid excretion processes and their accumulation in liver cells, as well as the development of fatty dystrophy (4). In addition, increased levels of circulating ketone bodies and acetone (substrates of CYP2E1) in the blood may cause an increase in CYP2E1 in the diabetic liver, leading to the development of insulin resistance. Insulin resistance, in turn, contributes to the maintenance of high levels of CYP2E1 and the progression of oxidative damage in hepatocytes (5). Insulin resistance also causes increased gluconeogenesis (6), and CYP2E1 plays a leading role in these processes. This contributes to an increase in glucose levels in liver cells and the development of hyperglycogenosis. In addition, ROS causes an increase in the expression of cytokines and stimulates inflammatory processes in the liver (7). Through the oxidation of fatty acids, CYP2E1 is also involved in *de novo* lipogenesis in fatty liver, whereby an interaction between CYP2E1 and PPARα-mediated fatty acid homeostasis has been demonstrated (8). The leading role of CYP2E1 in enhancing apoptotic processes and the development of liver fibrosis has been demonstrated (9). The demonstrated relationship between oxidative stress and the development of diabetic pathology provides a scientific basis for investigating the potential of using various natural compounds with antioxidant properties for the prevention and treatment of diabetic disease.

The antioxidant properties of omega-3 polyunsaturated fatty acids (PUFAs) have been demonstrated in animal disease models and in patients with cardiopathology and visceral organ pathology (10, 11). It should be noted that these compounds are substrates for CYP2E1 and may contribute to the accumulation of this protein in cells, which may increase the risk of oxidative stress. This should be considered when taking these compounds long-term, especially in diseases associated with high expression of CYP2E1, such as diabetic liver (4). In this work, we investigated the effect of long-term consumption of omega-3-enriched pharmaceutical composition of PUFAs on the level of CYP2E1 expression as a key factor in the initiation and development of liver pathology in diabetes mellitus. Prooxidant and antioxidant processes were also investigated, and the effect of omega-3 PUFAs on the ultrastructure of hepatocytes during the development of experimental diabetes was examined.

## Materials and methods

### Animal care and streptozotocin-induced diabetic rat model

Male Wistar rats (2 months old) were maintained in animal cages with free access to food and water on a 12/12-h light/dark cycle and room temperature. All animals were fed a standard diet. The non-diabetic control group included intact rats. Type 1 diabetes was induced by a single intraperitoneal injection of streptozotocin (STZ) at a dose of 50 mg/kg b.w.

The studies were conducted on three groups of animals: six non-diabetic control rats, six STZ-diabetic rats, and six STZ-diabetic rats treated with the pharmaceutical drug EPADOL (0.1 ml/100 g b.w. per day) purchased from Kyiv Vitamin Factory. EPADOL is the omega-3-enriched pharmaceutical composition of PUFAs. EPADOL contains ethyl esters of omega-3 PUFAs: 300 mg eicosapentaenoic acid (EPA) and 200 mg docosahexaenoic acid (DHA) per 1 ml. Thus, each animal received approximately 30 mg EPA and 20 mg DHA esters per 100 g b.w. per day.

STZ-diabetic rats were administered EPADOL by oral gavage once daily in the morning starting on the third day after STZ injection and for a period of 4 weeks. Blood glucose levels of all rats were checked three times: before injection of STZ, on the third day after injection (start of the experiment), and on the 28th day of the experiment (end of the experiment). A blood glucose level greater than 14 mmol/l on the third day after STZ injection was considered to indicate diabetes. Blood serum glucose levels were determined with an automated biochemical analyzer (Prestige 24i, Tokyo Boeki, Japan). The rats were decapitated under sodium pentobarbital anesthesia (60 mg/kg b.w.) at the end of the experiment. All manipulations of laboratory animals were performed in accordance with the European Convention for the Protection of Vertebrate Animals used for Experimental and other Scientific Purposes (Strasbourg, 1986). The protocol was approved by the Local Committee on Bioethics (registration number: 0114U007233).

### Western blot analysis and protein measurement

Preparation of liver samples for Western blot analysis and measurement of CYP2E1 protein levels were performed as previously described (12). CYP2E1 was identified using anti-CYP2E1 antibodies produced in Rabbit (Sigma-Aldrich, USA). The beta-actin (loading control) were visualized by mouse anti-beta-actin antibodies (Sigma-Aldrich, USA). Western blot analysis was carried out according to the manufacturer’s instruction for the use of antibodies. The treatment of membranes with secondary antibodies (Sigma-Aldrich, USA) was followed by chemiluminescence detection according to manufacturers’ instructions. Western blots were visualized and calculated using the ChemiDoc XRS+ system with Image Lab software (Bio-Rad, USA). Relative protein levels were calculated by comparing CYP2E1 levels with beta-actin levels and expressed as relative units.

### Measurement of monooxygenase activity of CYP2E1 in liver microsomes

Liver microsomes were obtained, and the p-nitrophenol (PNP) hydroxylase activity of CYP2E1 in microsomes was determined as previously described (3). Liver microsomes were obtained by ultracentrifugation at 100,000g for 60 min at 4°C. After ultracentrifugation, the microsomal pellet was suspended in storage buffer containing 100 mM Tris–HCl, pH 6.8, and 20% glycerol and aliquots were frozen at -70°C until needed. The reaction mixture for the measurement of the activity of CYP2E1 had the composition 1.0 mg of microsomal protein, 200 mM PNP (Sigma-Aldrich, USA) in 0.1 M potassium phosphate buffer (pH 6.8), and 1.0 mM ascorbic acid. The mixture was preincubated for 5 min at 37°C, and reaction was carried out with 1 mM NADPH (Sigma-Aldrich, USA) for 2 min at 37°C. The p-nitrocatechol (PNC) formed from PNP was detected by spectrophotometric measurement with absorbance of 546 nm. CYP2E1 activity values were expressed as nmol of p-nitrocatechol per minute per milligram of microsomal protein.

### Oxidative stress markers

Levels of lipid peroxidation (LPO), catalase, and superoxide dismutase (SOD) in liver tissue were determined as previously described (12). The level of LPO was determined in liver homogenates by assessing the level of malondialdehyde (MDA) as the main product of the reaction between thiobarbituric acid and lipid peroxides. Catalase activity was measured by hydrogen peroxide (H_2_O_2_) degradation. The reaction mixture consisted of 0.25% tissue homogenate, and 0.03% H_2_O_2_ (Sigma-Aldrich, USA) was incubated at 37°C for 10 min. The residual H_2_O_2_ was determined by adding ammonium molybdate and measured spectrophotometrically at 410 nm. SOD was assayed using nicotinamide adenine dinucleotide and phenazine methosulfate reagents for the reduction of nitro blue tetrazolium salt into blue-colored formazan measured spectrophotometrically at 560 nm.

The MDA values were expressed as mkmol per milligram of protein. The values of enzyme activities were expressed as U/mg protein (one unit of catalase activity means the amount of enzyme degrading 1 mkmol H_2_O_2_ per minute, one unit of SOD activity – the amount of enzyme oxidizing 1 nmol NADH per minute).

### Electron microscopic studies of rat livers

Preparation of liver tissues for microscopic studies of the ultrastructure of the liver was performed as previously described (3). For electron microscopic examination, 40–60-nm-thick ultrathin sections were contrasted with 1% uranyl acetate and lead citrate solution according to the method of Reynolds. The studies were performed using an electron microscope JEM 100CX (JEOL, Japan).

### Measurement of serum transaminases, gamma-glutamyl transferase levels, and lipid profile

The levels of activity of ALT, AST, and GGT in blood serum are useful biomarkers for liver pathology. A lipid profile is a test that measures the amount of total cholesterol, triacylglycerol (TAG), low-density lipoprotein cholesterol (LDL-C), and high-density lipoprotein cholesterol (HDL-C) in blood serum. Blood serum was obtained as previously described (3). The levels of activity of the enzymes and a lipid profile in blood serum were measured using an automated biochemical analyzer (Tokyo Boeki Prestige 24i, Japan, and HTI Biochem FC-200, USA).

### Statistical analysis

Statistical analysis was performed using Status software (http://status-please.herokuapp.com). Data were tested for normal distribution with Shapiro–Wilk test. Dispersion equality was tested using Levine test. Tukey-HSD test was used for multiple comparisons. Differences in means between groups were tested using one-way ANOVA and t-test. Statistically significant results were considered at p < 0.05. Results are presented as mean ± standard deviation (SD).

## Results

### Blood glucose levels and body weight of animals

All animals had physiological body weight values at the beginning of the experiment. At the end of the experiment, body weight values increased in the control group (27% compared to the time of the start of the experiment, *p* = 0.002), whereas the values remained unchanged in the diabetic group (**Table 1**).

**Table 1:** Body weight and glucose levels in the blood of the experimental animals.

Animals	Body weight, g		Blood glucose levels, mmol/L	
	Start of experiment	End of experiment	Start of experiment	End of experiment
Non-diabetic rats	172.0 ± 17.175	218.0 ± 14.404*	8.08 ± 0.228	8.14 ± 0.329
STZ-diabetic rats	162.14 ± 11.495	162.86 ± 24.640	25.91 ± 3.765 ^♦^	31.91 ± 2.042 ^♦^
STZ-diabetic rats administered with omega 3 PUFAs	178.83 ± 9.806	181.17 ± 18.280	22.97 ± 1.663 ^♦^	29.02 ± 2.941 ^♦^

Values are means ± SD, n = 6 rats in each group. *p ≤ 0.01 compared to the start of the experiment (Student’s t-test), ^♦^p ≤ 0.001 compared to the nondiabetic group (one-way ANOVA).

Before the start of the experiment, the blood glucose levels of all intact animals were within the physiological range (approximately 8 mmol/l). In the diabetic group, a significant increase in blood glucose levels (three-fold compared to the intact control group, *p* = 0.001) was observed on the third day after injection of STZ. At the end of the experiment, glucose levels were four times higher than in the intact control (*p* = 0.001) (**Table 1**).

Treatment with omega-3 PUFAs did not decrease glucose levels in the diabetic rats. These levels remained elevated by more than 3.5-fold compared with non-diabetic controls (*p* = 0.001) (**Table 1**). No changes in body weight were observed in the diabetic rats administered with omega-3 PUFAs during the experiment (**Table 1**).

### CYP2E1 activity and protein levels in the liver

**Figure 1:**
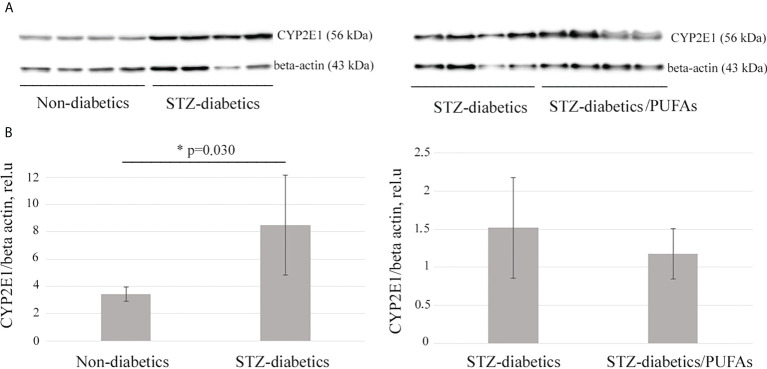
CYP2E1 protein levels in the liver of experimental rats. **(A)** Western blot analysis of total liver lysates probed with specific anti-CYP2E1 antibodies. Beta-actin is a loading control. **(B)** Quantification of Western blotting results. *p-values < 0.05 were considered statistically significant (Student’s t-test). Means ± SD (n = 4 in each group).

**Figure 2:**
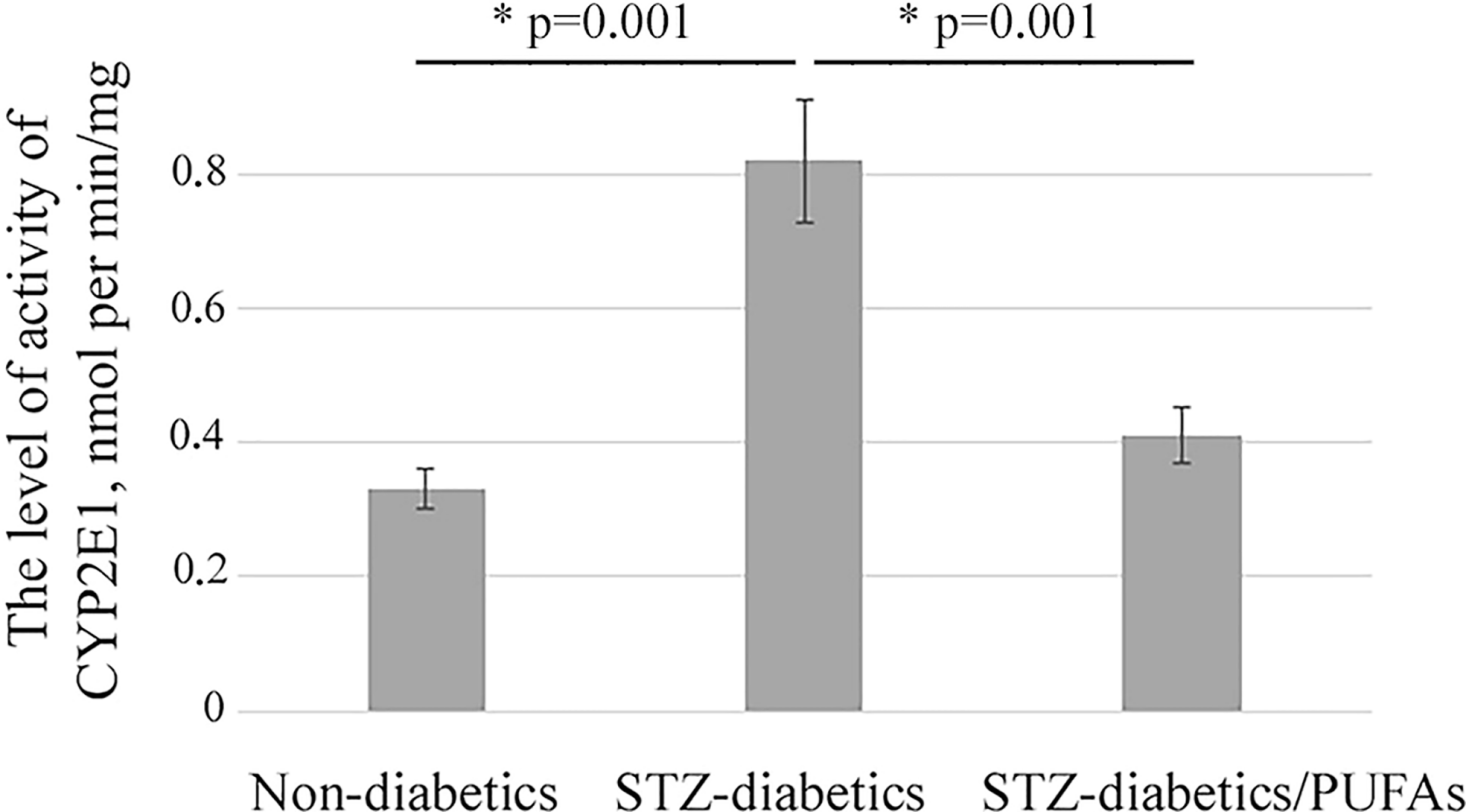
p-Nitrophenol hydroxylase activity of CYP2E1 in microsomes from liver of experimental animals; *p-values < 0.05 were considered statistically significant (one-way ANOVA). Means ± SD (n = 4-6 in each group).

A 2.5-fold increase in CYP2E1 protein level was observed in the liver of STZ-diabetic rats compared to the non-diabetic control (*p* = 0.030) (**Figure 1**). It was also found that an increase in the enzyme level resulted in a more than two-fold increase in its monooxygenase activity (*p* = 0.001) (**Figure 2**).

It was found that administration of omega-3 PUFAs to diabetic rats did not result in a significant change in CYP2E1 levels compared to the STZ-diabetic group (*p* = 0.388) (**Figure 1**). At the same time, CYP2E1 activity was significantly decreased (two-fold) compared to the diabetic group (*p* = 0.001) and approached the levels of the non-diabetic control group (*p* = 0.192) (**Figure 2**).

### Oxidative stress markers in the liver of STZ-induced diabetic rats

**Figure 3:**
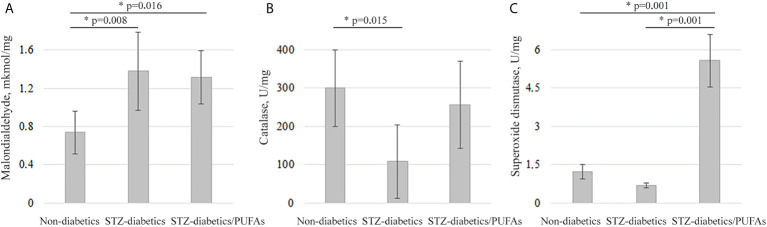
Oxidative stress markers in the liver tissues of the experimental animals. **(A)** Malondialdehyde levels, **(B)** catalase activity, **(C)** superoxide dismutase activity. *p-values < 0.05 were considered statistically significant (one-way ANOVA). Means ± SD (n = 6 in each group).

Previously, it was shown that an increase in CYP2E1 expression can lead to the development of oxidative stress in cells (4). We found the signs of this stress in the liver of experimental diabetic animals. A 1.9-fold increase in malondialdehyde levels (*diabetic vs. control, p* = 0.008) was observed, which may indicate an intensification of lipid peroxidation processes. At the same time, a depletion of the antioxidant system was observed. Specifically, catalase and SOD activities were decreased 2.8-fold and 1.8-fold, respectively, in the livers of diabetic animals compared to non-diabetic controls, but for SOD there were no statistically significant data (catalase, *p* = 0.015; SOD, *p* = 0.312) (**Figure 3**).

It was found that treatment with omega-3 PUFAs did not decrease peroxide processes, which remained close to the diabetic group. At the same time, the levels of catalase and SOD activity increased by 2.4-fold and 8-fold, respectively, compared to the diabetic group, but for catalase there were no statistically significant data (catalase, *p* = 0.062; SOD, *p* = 0.001). Catalase activity remained at the control level, and the SOD activity was 4.5-fold higher than in the non-diabetic control group (*p* = 0.001). Thus, no signs of oxidative stress were detected in the livers of diabetic rats consuming omega-3 PUFAs (**Figure 3**).

### Electron microscopic studies of the livers of experimental animals

In our recent study, the structural characteristics of the livers of intact as well as STZ-diabetic male Wistar rats were described. No signs of liver pathology were detected in the liver samples of the intact rats (3). At the same time, we found significant ultrastructural changes (structural and functional damage to organelles, especially mitochondria, as well as signs of disturbances in metabolic processes, including fat dystrophy) in the livers of the diabetic animals (3).

**Figure 4:**
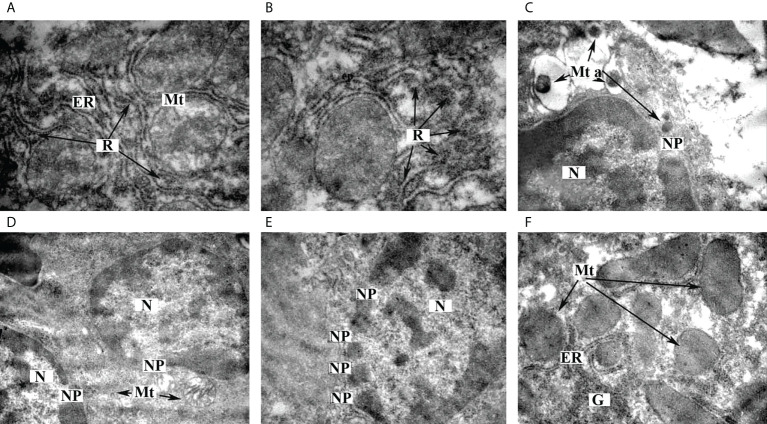
Ultrastructural features of hepatocytes from STZ-diabetic rats administered with omega-3 PUFAs. **(A)** Ultrastructure of endoplasmic reticulum, ×12,000. **(B)** Localization of ribosomes on the ER membrane, ×12,000. **(C)** Ultrastructure of apoptotic mitochondria, ×6,400. **(D)** Ultrastructure of nucleus, ×6,400. **(E)** Transport of informational and protein molecules between the nucleus and cytoplasm of hepatocyte, ×8,000. **(F)** Glycogen granules in hepatocytes, ×8,000. *ER*—endoplasmic reticulum, *G*—glycogen granules, *Mt*—mitochondria, *Mt a*—apoptotic mitochondria, *N*—nucleus, *NP*—nuclear pores, *R*—ribosomes.

In this work, we investigated the ultrastructural features of liver tissue from diabetic rats treated with omega-3 PUFAs. We found a marked granularity of the endoplasmic reticulum, which may indicate an increase in protein synthesis in hepatocytes (**Figures 4A, B**). Different forms of mitochondria were detected: most of the organelles showed signs of normal adult functional forms with vesicular cristae (**Figure 4A**), some of the organelles showed signs of young forms with an electron-dense matrix without clearly defined cristae (**Figure 4E**), and some of the mitochondria showed apoptotic changes (**Figure 4C**) and destructive changes (**Figure 4D**). The presence of different forms of mitochondria with a predominance of functionally active mitochondria may indicate optimal energy production in cells. The formation of a large number of nuclear pores of considerable size (**Figures 4C–E**) was found, probably indicating the activation of the processes of transport of informational and protein molecules across the nuclear membrane. This may also be evidenced by the location of mitochondria in the perinuclear region (**Figures 4C, D**). A considerable number of glycogen granules were found, some of which are collected in rosettes (**Figure 4F**).

Thus, in the present work, we have shown that treatment with omega-3 PUFAs prevents damage to the ultrastructure of the liver of diabetic rats, namely, structural and functional damage to organelles, especially mitochondria and the nucleus, which we had previously demonstrated in liver samples from diabetic rats (3). Biosynthetic processes and the processes of transport of molecules across the nuclear membrane were significantly enhanced. There were also signs of normal function of the mitochondrial apparatus. It should be noted that no lipid granules were found in the samples studied, which may indicate the prevention of the development of lipid dystrophy, which often occurs in diabetic liver. However, there were signs of impaired carbohydrate metabolism in the liver samples (**Figure 4**).

### Serum markers of a liver destruction STZ-induced diabetic rat model

**Figure 5:**
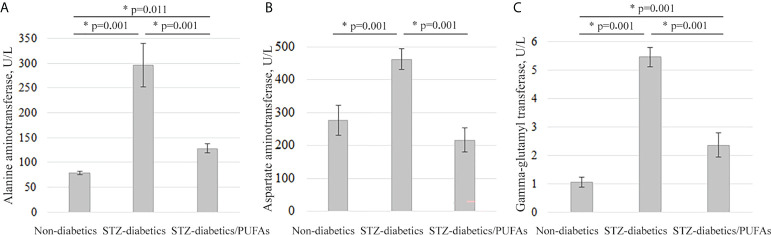
Serum markers of liver destruction in the experimental animals. **(A)** Alanine aminotransferase levels, **(B)** aspartate aminotransferase levels, and **(C)** gamma-glutamyl transferase levels. *p-values < 0.05 were considered statistically significant (one-way ANOVA). Means ± SD (n = 6 in each group).

The signs of liver pathology were found in the blood serum of the diabetic rats. We discovered increased activity of transaminases: ALT (by 3.8-fold), AST (by 1.7-fold), and GGT (by more than 5-fold) compared to control (ALT, *p* = 0.001; AST, *p =* 0.001; GGT, *p* = 0.001) (**Figure 5**).

It was found that the levels of ALT, AST, and GGT activity in the blood serum of the diabetic animals administered with omega-3 PUFAs were significantly lower (approximately by 2-fold) than in the diabetic group (ALT, *p* = 0.001; AST, *p =* 0.001; GGT, *p* = 0.001). Thus, AST approached control values, while ALT and GGT remained elevated compared to the non-diabetic control group (ALT, *p* = 0.011; AST, *p* = 0.060; GGT, *p* = 0.001) (**Figure 5**).

### Serum lipid profile of STZ-induced diabetic rats

The lipid profile abnormality (dyslipidemia) is a common symptom of diabetes, as well as a risk factor for non-alcoholic fatty liver disease (13, 14). Some signs of dyslipidemia were found in the blood serum of the diabetic rats. We discovered increased levels of the triacylglycerol (68%) and decreased level of the high-density lipoprotein cholesterol (39%) compared to non-diabetic control (TAG, *p* = 0.013; HDL-C, *p* = 0.001). The levels of total cholesterol and low-density lipoprotein cholesterol approached control values (cholesterol, *p* = 0.200; LDL-C, *p* = 0.179) (**Table 2**).

**Table 2:** Serum lipid profile of experimental animals.

Animals	Total cholesterol, mmol/L	Triacylglycerol, mmol/L	High-density lipoprotein cholesterol, mmol/L	Low-density lipoprotein cholesterol, mmol/L
Non-diabetic rats	1.09 ± 0.304	0.57 ± 0.029	0.46 ± 0.085	0.74 ± 0.288
STZ-diabetic rats	0.80 ± 0.240	0.95 ± 0.238 *	0.28 ± 0.008 *	0.49 ± 0.218
STZ-diabetic rats administered with omega 3 PUFAs	1.00 ± 0,266	1.14 ± 0.259 *	0.28 ± 0.021 *	0.58 ± 0.149

Values are means ± SD, n = 6 rats in each group. *p ≤ 0.01 compared to the non-diabetic group (one-way ANOVA).

It was found that the cholesterol, TAG, LDL-C, and HDL-C levels in the blood serum of the diabetic animals administered with omega-3 PUFAs did not change compared to the diabetic group (cholesterol, *p* = 0.436; TAG, *p =* 0.287; LDL-C, *p* = 0.795; and HDL-C, *p* = 0.900). Thus, the treatment with omega-3 PUFAs did not contribute to the normalization of the lipid profile (*diabetics/PUFAs vs. non-diabetic control*, cholesterol, *p* = 0.842; TAG, *p* = 0.001; LDL-C, *p* = 0.444; HDL-C, *p* = 0.001) (**Table 2**).

## Discussion

Due to their antioxidant, anti-inflammatory, antiapoptotic, and other beneficial properties, omega-3-enriched pharmaceutical compositions of PUFAs are widely used for the prevention of the pathologies of the cardiovascular and endocrine systems, especially in diabetes mellitus (11). It should be noted that the systemic metabolism of PUFAs entering the body from the outside occurs mainly in the liver (15). Enzymes of the cytochrome P450 superfamily, especially CYP2E1, play the main role in the metabolism of omega PUFAs (16). Omega-3 PUFAs, as substrates, can increase the expression level of CYP2E1 in cells. We have recently shown that a diet enriched with omega-3 PUFAs leads to an increase in CYP2E1 levels in the liver of young healthy animals (12). It has also been shown that an increase in CYP2E1 expression can lead to oxidative stress, oxidative ultrastructural damage, and the development of pathology (4). Therefore, the question arises whether long-term treatment with CYP2E1 substrates may increase the risk of developing CYP2E1-dependent oxidative stress in some diseases (including diabetes) whose pathogenesis is closely associated with increased expression of CYP2E1. Our work aimed to address this question.

In our experiment, we detected an increase in the level and activity of CYP2E1 in the liver of diabetic rats. Several factors could lead to this, including a decrease in the inhibitory effect of insulin on gene transcription, substrate stabilization of CYP2E1 molecules by acetone and ketone bodies, and intensification of gluconeogenesis with active participation of CYP2E1 (17). Such increased expression of CYP2E1 could cause the development of oxidative stress, the signs of which were found in diabetic liver in the present study. In our previous work, we found that CYP2E1-dependent oxidative stress caused ultrastructural damage in the liver tissue of diabetic animals, which was accompanied by a significant increase in liver damage biomarkers in serum (12). In the present work, liver tissue destruction was also evidenced by an increase in serum markers of liver pathology. Our previous ultrastructural studies have found signs of fatty liver in diabetic rats (12). It is known that defects in insulin action, increased concentrations of free fatty acids, and non-infectious inflammation lead to the disturbed regulation of lipoprotein metabolism in the liver, resulting in an abnormal serum lipid profile in diabetics (14). It was shown that dyslipidemia is a risk factor for non-alcoholic fatty liver disease (15). It was found that CYP2E1-mediated mechanisms may be involved in such processes (7, 8). We have identified signs of dyslipidemia, namely, an increase in TAG level and a decrease in HDL-C level. A similar lipid profile abnormality was found in patients with type 1 diabetes (18).

We investigated the level of CYP2E1 expression (as a key factor in the development of pathology) in the liver of diabetic rats treated with omega-3 PUFAs. We found no changes in the level of the enzyme but noted a significant decrease in its activity compared to the non-treated diabetic group. Such maintenance of a high CYP2E1 content may be associated with a high level of substrates that stabilize the enzyme molecule and protect it from rapid degradation. At the same time, a high level of CYP2E1 substrates may cause the effect of substrate inhibition (19), which we observed when studying the p-nitrophenol hydroxylase activity of the enzyme *in vitro*. The increased expression of CYP2E1 may be the reason for the high peroxide processes we observed in the liver of diabetic rats treated with omega-3 PUFAs. It was shown that increased CYP2E1 expression and CYP2E1-dependent oxidative processes can lead to the development of fatty dystrophy (4). In our recent studies, we observed signs of lipid metabolism disorders in the liver of diabetic rats (3). In the present work, administration of omega-3 PUFAs led to some activation of antioxidant enzymes, which could prevent oxidative damage to the liver. The ability of omega-3 PUFAs to activate antioxidant resources has also been demonstrated by other authors (11). It was shown that omega-3 fatty acid treatment may have beneficial effects in regulating hepatic lipid metabolism (20). Indeed, we found no signs of fatty liver. However, the serum lipid profile did not normalize. Dyslipidemia is due to insulin dysregulation and hyperglycemia (14), and omega-3 PUFAs do not affect these processes.

Thus, consumption of omega-3 PUFAs was shown to prevent oxidative damage to liver tissue in diabetic rats. This contributed to the maintenance of the processes of biosynthesis, energy metabolism, and lipid metabolism in liver cells. The absence of significant structural and functional disorders of the liver of diabetic rats treated with omega-3 PUFAs can be demonstrated by the data of microscopic ultrastructural analysis and biochemical analysis of the level of serum markers of liver pathology.

It should be noted that significant accumulations of glycogen in liver cells and hyperglycemia were also detected in the present work. These data may indicate inhibition of the processes of glucose entry into the liver and stimulation of gluconeogenesis in the cells, with an increase in CYP2E1 levels playing a leading role in the activation of this process. Thus, it was found that treatment with omega-3 PUFAs did not normalize carbohydrate metabolism in the liver of diabetic rats.

## Conclusion

We detected a significant increase in CYP2E1 expression (protein content and activity) and found evidence of oxidative stress, which may have been induced by a high CYP2E1 concentration in the liver of diabetic rats. The significant increase in blood serum transaminases detected could indicate marked destruction of liver tissue. All these signs could indicate the development of diabetic pathology in the liver of the experimental animals. Significant hyperglycemia and diabetic dyslipidemia (increased triacylglycerol levels and decreased HDL-C levels) were found. Lack of natural weight gain in the rats was also noted.

Treatment of the diabetic animals with an omega-3-enriched pharmaceutical composition of PUFAs had no effect on CYP2E1 levels, which remained at the diabetic levels, but contributed to a decrease in enzyme activity. The high level of CYP2E1 could be the reason for the high level of peroxide processes. However, due to the induction of antioxidant activity, treatment with omega-3 PUFAs may prevent oxidative damage to the liver. Examination of the ultrastructure of liver tissues and cells revealed no signs of diabetic pathology, with the exception of hyperglycogenosis. The markedly increased accumulation of glycogen granules in the hepatocytes, as well as the significant hyperglycemia, might indicate that the omega-3 PUFAs have no effect on carbohydrate metabolism. We also found no signs of fatty liver disease. However, the serum lipid profile did not normalize.

Thus, the results obtained might indicate that long-term treatment of diabetic animals with omega-3 PUFAs does not increase the risk of CYP2E1-dependent oxidative stress and the development of liver pathology but rather prevents some diabetic ultrastructural damage to hepatocytes.

## Data availability statement

The original contributions presented in the study are included in the article/Supplementary Material. Further inquiries can be directed to the corresponding author.

## Ethics statement

The animal study was reviewed and approved by The Local Committee on Bioethics (registration number: 0114U007233), Bogomoletz Institute of Physiology, National Academy of Sciences of Ukraine, Kyiv, Ukraine.

## Author contributions

OM and AS contributed equally to this work and share first authorship. OM and AS made substantial contributions to the conception and design, as well as acquisition and analysis of data, and participated in data analysis and critical review of the manuscript for important intellectual content. OM performed Western blot analysis and CYP2E1 activity measurements. AS examined markers of oxidative stress. DS conducted statistical analysis. The first draft of the manuscript was written by OM. All authors contributed to the revision of the manuscript and read and approved the submitted version.

## Funding

The authors declare that this study received funding from the National Research Foundation of Ukraine under the project “*Support* for *Research of Leading and Young Scientists*” (No. 20220.02/0332). The funder was not involved in the study design, collection, analysis, interpretation of data, the writing of this article, or the decision to submit it for publication.

## Acknowledgments

The authors thank Dr. Katherine Rozova (Bogomoletz Institute of Physiology, National Academy of Sciences of Ukraine) for valuable help with microscopic studies. The authors thank Academic Proofreading (https://www.academicproofreading.uk/) for their significant contribution to the English language editing of the manuscript.

## Conflict of interest

The authors declare that the research was conducted in the absence of any commercial or financial relationships that could be construed as a potential conflict of interest.

## Publisher’s note

All claims expressed in this article are solely those of the authors and do not necessarily represent those of their affiliated organizations, or those of the publisher, the editors and the reviewers. Any product that may be evaluated in this article, or claim that may be made by its manufacturer, is not guaranteed or endorsed by the publisher.
